# Effect of Hyaluronan in Collagen Biomaterials on Human Macrophages and Fibroblasts *In Vitro*

**DOI:** 10.3390/jfb16050167

**Published:** 2025-05-08

**Authors:** Nancy Avila-Martinez, Maren Pfirrmann, Madalena L. N. P. Gomes, Roman Krymchenko, Elly M. M. Versteeg, Marcel Vlig, Martijn Verdoes, Toin H. van Kuppevelt, Bouke K. H. L. Boekema, Willeke F. Daamen

**Affiliations:** 1 Department of Medical BioSciences, Research Institute for Medical Innovation, Radboud university medical center, 6525 GA Nijmegen, The Netherlands; 2 Department of Pathology, Amsterdam University Medical Center (AUMC), Location AMC, 1081 HV Amsterdam, The Netherlands; 3 Tissue Function and Regeneration, Amsterdam Movement Sciences Research Institute, 1081 HV Amsterdam, The Netherlands; 4 Department of Plastic, Reconstructive and Hand Surgery, Amsterdam University Medical Center (AUMC), Location AMC, 1081 HV Amsterdam, The Netherlands; 5 Alliance of Dutch Burn Care, Burn Research Lab, 1941 AJ Beverwijk, The Netherlands; 6 Department of Immunology, Leiden University Medical Center (LUMC), 2333 ZA Leiden, The Netherlands

**Keywords:** hyaluronic acid, scaffolds, skin regeneration, dermal, fetal, eschar

## Abstract

In adults, scars are formed after deep skin wound injuries like burns. However, the fetal microenvironment allows for scarless skin regeneration. One component that is abundantly present in the fetal extracellular matrix is hyaluronan (HA). To study whether biomaterials with HA improve wound healing, type I collagen scaffolds with and without HA were prepared and characterized. Their immune effect was tested using macrophages and their phenotypes were analyzed through cell surface markers and cytokine expression after 48 h. Since fibroblasts are the main cellular component in the dermis, adult, fetal and eschar-derived cells were cultured on scaffolds for 14 days and evaluated using histology, gene and protein expression analyses. Biochemical assays demonstrated that HA was successfully incorporated and evenly distributed throughout the scaffolds. Macrophages (M0) cultured on Col I+HA scaffolds exhibited a profile resembling the M2c-like phenotype (CD206^high^, CD163^high^ and IL10^high^). HA did not significantly affect gene expression in adult and fetal fibroblasts, but significantly reduced scarring-related genes, such as transforming growth factor beta 1 (TGFB1) and type X collagen alpha 1 chain (COL10A1), in myofibroblast-like eschar cells. These findings highlight the potential of incorporating HA into collagen-based skin substitutes to improve the wound healing response.

## 1. Introduction

Effective skin wound healing is vital for quality of life but remains a significant global medical challenge [1]. Standard care for full-thickness burns includes autologous split-thickness skin grafting, which replaces the epidermis and part of the dermis [2]. Biocompatible skin substitutes, particularly natural biomaterials like collagen, emerged in the wound care market as they support dermal restoration. From the premise that fetal wounds can heal without scars, researchers have widely explored hyaluronan, an abundant component of the fetal extracellular matrix [3,4,5].

Hyaluronan (HA), also known in its protonated form as hyaluronic acid, is a non-sulfated glycosaminoglycan that carries a significant overall negative charge and contains carboxylate and acetamido functional groups [6]. HA has a remarkable capacity to bind and retain water, which results in the moisturization of the dermal extracellular matrix. During the wound healing process, HA binds primarily to the CD44 receptor on the cell surface, facilitating cell adhesion and migration [7].

During hemostasis and inflammation, circulatory monocyte-derived macrophages (M0) migrate to the wound site, gradually replacing short-lived neutrophils [8]. Macrophages serve as important regulators as they can polarize into M1type macrophages, which secrete pro-inflammatory cytokines during proliferation. A persistent response from this phenotype might lead to more scarring. M1s are later replaced by M2-type macrophages, which release anti-inflammatory/pro-healing cytokines in the remodeling stage, making them key targets for improving wound healing outcomes [9]. Macrophages have a high CD44 expression and play a major role in HA uptake and degradation, where the M1-like subtype favors HA binding and the M2-like subtype shows improved HA internalization [10].

Fibroblasts are important cells in the proliferation and remodeling phases of wound healing, but their behavior depends on the life stage of the patient and their cellular origin. For instance, adult fibroblasts showed a four times lower CD44 expression compared to their fetal equivalent [11]. Additionally, adult wounds showed increased transforming growth factor beta 1 (TGFβ1) expression, with low levels of TGFβ3, whereas an opposite pattern was observed in fetal wounds [12]. The role of cellular origin is exemplified by adult eschar fibroblasts obtained from burnt tissue after debridement. These eschar fibroblasts have been associated with a myofibroblast-like phenotype due their expression of alpha smooth muscle actin (α-SMA) and ability to contract collagen scaffolds in vitro [13].

Several studies have indicated the benefits of using HA in wound dressings and its effect on skin cells, such as dermal fibroblasts and macrophages [14,15]. While commercial HA-based products are mainly available in cream or gel form, porous sponges offer distinct advantages, especially for full-thickness wounds, such as a larger surface area for cell attachment [16,17]. In this study, HA was chemically crosslinked to porous collagen scaffolds and compared to bare collagen scaffolds to evaluate the effect of HA addition on primary cells, such as on macrophage polarization, as well as on particular fibrotic gene expression levels in fetal, healthy adult and adult eschar fibroblasts.

## 2. Materials and Methods

### 2.1. Preparation and Characterization of Type I Collagen Scaffolds with Hyaluronan

Porous collagen scaffolds (Col I) were prepared by adding 0.8% (*w*/*v*) collagen fibrils from bovine tendon, obtained from a slaughterhouse, to 0.25 M acetic acid and swelling overnight at 4 °C under constant agitation. For collagen scaffolds with hyaluronan (Col I+HA), 0.05% (*w*/*v*) hyaluronic acid sodium salt from *Streptococcus equi* (Sigma-Aldrich, St. Louis, MO, USA) was dissolved in 0.25 M acetic acid, and 0.8% (*w*/*v*) type I collagen fibrils were added. The suspension was mixed, swollen overnight at 4 °C, homogenized, poured into molds (4 mL suspension/960 mm^2^, 6-well plate) and lyophilized using a Lyoph-pride 03 freeze dryer (ilShin Biobase Europe, Ede, The Netherlands). Scaffolds were chemically crosslinked for 3 h using 33 mM 1-ethyl-3-dimethyl aminopropyl carbodiimide (EDC) and 6 mM N-hydroxysuccinimide (NHS) in 50 mM 2-morpholinoethane sulphonic acid buffer (Sigma-Aldrich) containing 40% ethanol (Merck, Darmstadt, Germany) at pH 5.0 (MES buffer) (8 mg scaffold/mL crosslinking solution). Non-crosslinked control scaffolds were treated similarly, but without EDC/NHS. Scaffolds were washed in 0.1 M Na_2_HPO_4_ (2 × 1 h), 1 M NaCl (2 × 30 min), 2 M NaCl (2 × 30 min) and demineralized water until their conductivity was less than 200 µS, and lyophilized. Scaffolds were cut to ∅12 mm for in vitro studies.

The morphology of the scaffolds was analyzed using scanning electron microscopy (SEM). Dry scaffolds were fixed on a stub with double-sided carbon tape, sputtered with gold twice for 60 s using an Edwards Scancoat Six Sputter Coater (Crawley, UK) and examined with a Zeiss Sigma 300 Field Emission Scanning Electron Microscope (Zeiss, Jena, Germany) at an accelerating voltage of 3 kV.

For HA localization, frozen scaffolds in TissueTek O.C.T. compound (Sakura Finetek, Alphen aan den Rijn, The Netherlands) were cut to 7 µm sections and mounted on Superfrost™ plus adhesion microscope slides (Epredia, Kalamazoo, MI, USA). After 10 min fixation in 4% paraformaldehyde in phosphate-buffered saline (PBS), sections were blocked with PBS containing 0.5% bovine serum albumin (BSA, fraction V, Roche, Basel, Switzerland) and stained with biotinylated HA binding protein (400763-1A, Seikagaku, Tokyo, Japan), 1:200 diluted in 0.5% BSA in PBS. The signal was amplified by incubating the sample with the Vectastain avidin/biotin ABC complex HRP kit (PK-4000, Vector Laboratories, Newark, CA, USA) for 1 h. Finally, ImmPACT AEC (3-amino-9-ethylcarbazole) was added to produce a red reaction product over 15 min, followed by rinsing with water for 5 min. The sections were mounted with a cover glass using VectorMount^®^ AQ Aquos Mounting Medium (H-5501, Vector Laboratories).

The degree of crosslinking was determined by the loss of primary amine groups after crosslinking using 2,4,6-trinitrobenzene sulfonic acid (TNBS) [18]. Briefly, scaffolds before and after crosslinking were incubated in 4% NaHCO_3_, left to react with 0.08% (*w*/*v*) TNBS for 2 h at 40 °C and hydrolyzed in 6 M HCl for 1.5 h at 60 °C. Glycine was used for the calibration curve. The absorbances were measured at 420 nm using a SpectraMax iD3 spectrophotometer (Molecular Devices, San Jose, CA, USA).

The HA content in the scaffolds was measured using the Stains-All colorimetric assay using a carbocyanine dye suitable for staining HA (Sigma-Aldrich). Scaffolds were digested overnight at 65 °C using 2.5 U/mL papain (P3125, Merck), 2 mM EDTA and 2 mM cysteine in 50 mM sodium phosphate (pH 6.5). A staining solution, containing 0.0056% (*w*/*v*) Stains-All dissolved in 5% (*v*/*v*) isopropanol (Merck), 5% (*v*/*v*) 0.01 M ascorbic acid (Sigma-Aldrich) and 0.2% (*v*/*v*) 1 M acetic acid (Merck) in demineralized water, was added to the digested samples alongside a calibration curve of HA, and the absorbances were measured at 650 nm.

The molecular weight of the incorporated HA in the scaffolds was estimated using agarose gel electrophoresis. Briefly, 1% LE agarose (SeaKem, Lonza, Rockland, ME, USA) was dissolved in TAE buffer (40 mM Tris, 20 mM acetic acid, 1 mM EDTA, pH 8.0), heated and poured into a tray. Papain-digested collagen scaffolds with and without HA (as described in HA quantification), along with undigested HA and papain-digested HA, were loaded on the gel, and Smart Ladder (MW-1700-10, Eurogentec, Seraing, Belgium) and Lambda DNA/Hind III marker (G171A, Promega, Madison, WI, USA) were used as the molecular weight standards. Their base pair size was converted to molecular weight (MW) size using bioline.com to assess HA size in the gel. The gel was run for ~2 h at 50 V and was fixed for 2 × 1 h in 25% (*v*/*v*) isopropanol and 10% (*v*/*v*) acetic acid in demineralized water. The gel was stained overnight in the dark under constant agitation with 0.01% (*w*/*v*) Stains-All, 7.5% (*v*/*v*) formamide and 25% (*v*/*v*) isopropanol in 4 mM Tris pH 8.8 and destained with 25% isopropanol for two days, changing the solution four times. The gel was scanned using the Gel Doc XR+ Imaging System (Bio-Rad, Hercules, CA, USA).

For the differential scanning calorimetry assay (DSC), approximately 1 mg of sample was placed in T_zero_ pans (TA Instruments, New Castle, DE, USA), which were tightly sealed with T_zero_ hermetic lids, and heated from 1 to 220 °C at a scanning rate of 5 °C/min using a TA Q1000 DSC (TA Instruments, New Castle, WA, USA) equipped with a RCS40 cooler with Universal Analysis software version 5.5.24. T_onset_ was selected to detect the denaturation temperature of the collagen peaks [19].

### 2.2. Macrophage Culture and Analysis

**Figure 1:**
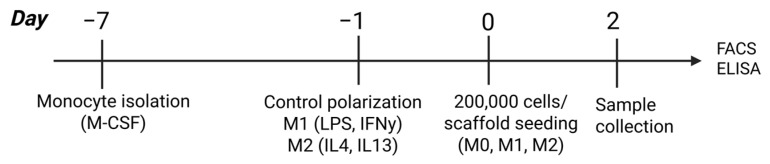
Timeline for macrophage culture on scaffolds. Analyses performed after two days and included fluorescence-activated cell sorting (FACS) and enzyme-linked immunoassay (ELISA). Created in BioRender, https://BioRender.com/i2p6ff7 (accessed on 20 April 2025).

Peripheral blood mononuclear cells (PBMCs) were isolated from buffy coats from three healthy donors (Sanquin, Nijmegen, The Netherlands). Informed consent was given in accordance with the Declaration of Helsinki and the Dutch national and Sanquin internal ethics boards. Monocytes were isolated using CD14^+^ MACS MicroBeads (130-050-201, Miltenyi Biotech, Teterow, Germany) following the manufacturer’s protocol. Monocytes were plated in T75 flasks (10^6^ cells in 10 mL for M0 differentiation and 0.5 × 10^6^ cells for M1/M2 differentiation) in RPMI 1640 (including HEPES, Gibco by Thermo Fisher Scientific, Grand Island, NY, USA), 10% fetal bovine serum (FBS, Gibco), 1% stable glutamine (STA-B, Capricorn, Ebsdorfergrund, Germany) and 1% antibiotic–antimycotic (Gibco), and differentiated with 50 ng/mL macrophage colony-stimulating factor (M-CSF, 300-25, PeproTech by Thermo Scientific, Waltham, MA, USA) for 6 days to obtain monocyte-derived macrophages. The macrophages were either left unstimulated (M0 macrophages) or were stimulated for 24 h with 100 ng/mL lipopolysaccharides (LPS, vac-3pelps, InvivoGen, San Diego, CA, USA) + 20 ng/mL interferon gamma (IFNγ, 300-02, PeproTech) to polarize them to M1-like macrophages, or treated with 20 ng/mL interleukin 4 (IL4, 170-076-135, Miltenyi Biotec) + 20 ng/mL interleukin 13 (IL13, 130-112-412, Miltenyi Biotech) to polarize them to M2-like macrophages. On day 7, the cells were collected after 30 min of incubation with PBS and 2 mM EDTA. A total of 200,000 cells were seeded on each scaffold and cultured for 48 h (Figure 1). M0 macrophages were seeded on Col I and Col I+HA scaffolds to observe their polarization, while M1-like and M2-like macrophages were seeded separately in Col I scaffolds as controls.

For flow cytometric analysis, cells were isolated from scaffolds by incubation with 0.25 U/mL collagenase A (10103578001, Roche, Mannheim, Germany) at 37 °C in a shaking water bath for 30 min. All the following incubations were performed at 4 °C. PBS with 2 mM EDTA was added to cell-seeded scaffolds and incubated for 30 min under constant agitation, filtered using a 70 μm cell strainer (Falcon, Durham, NC, USA), centrifuged at 257× *g* for 10 min and transferred to a 96-well V-bottom plate for staining. Isolated cells were incubated with eFluor 780-APC+Cy7, dilution 1:2000 (65-0865-14, ThermoFisher, Carlsbad, CA, USA), for 20 min in the dark and washed with PBA (PBS +1% BSA + 0.05% NaN_3_) for 25 min. CD163, CD206, CD80, MerTK, HLA-DR and PDL1 antibodies were used for labeling (Supplementary Table S1). Marker expression was measured with the BD FACSVerse Cell Analyzer flow cytometer (BD BioSciences, Franklin Lakes, NJ, USA). Data analysis was performed using FlowJo X (vX 0.7, Tree STAR, Ashland, OR, USA). Cells were gated on live cells and CD45^+^ cells, and mean fluorescence intensity (MFI) for each marker was assessed (Supplementary Figure S1).

ELISA kits for IL12 (88-7126), IL6 (88-7066) and IL10 (88-7106) (Invitrogen, ThermoFisher, Vienna, Austria) were used to measure the concentrations of cytokines in the supernatants according to the manufacturer’s instructions. Supernatants from M1 control were diluted 10×. The absorptions were measured at 450 nm using the iMark™ Microplate Absorbance Reader (Bio-Rad, Basel, Switzerland).

### 2.3. Fibroblast Culture and Analysis

Fibroblasts were obtained from three different sources, either healthy adult skin (adult), burn wound tissue (eschar) or fetal skin (fetal), as described [20,21]. Cells were isolated from three different donors per tissue type. In total, nine donors were tested. Adult fibroblasts were isolated from healthy skin samples obtained from adult patients who underwent elective surgery at the Department of Plastic and Reconstructive Surgery of the Red Cross Hospital (Beverwijk, NL). Eschar material was obtained from patients undergoing debridement between day 13 and 19 post-burn as part of their treatment in the Burn Center of the Red Cross Hospital. Fetal fibroblasts were isolated from fetal skin after pregnancy termination between gestational weeks 16 and 22. Fetal skin was obtained with the informed and written consent of the pregnant women from the Center for Contraception, Abortion and Sexuality (CASA, Leiden and The Hague, NL). Consent for the use of anonymized residual tissue was received through the informed opt-out protocol in accordance with national guidelines (https://www.coreon.org/, accessed on 23 November 2020) and was approved by the institutional privacy officer.

Cells were cultured in fibroblast medium (FBM) including DMEM with 10% fetal bovine serum (FBS), 1% penicillin/streptomycin and 1% GlutaMAX (all from Gibco, Paisley, UK). Cells were incubated at 37 °C with 5% CO_2_.

**Figure 2:**
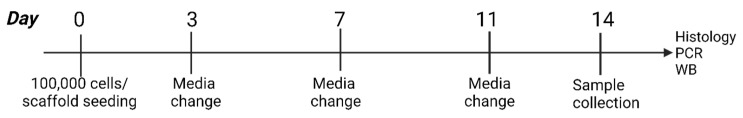
Timeline for fibroblast culture on scaffolds. Analyses performed on day 14 and included histology using H&E staining, real-time quantitative polymer chain reaction (PCR) and Western blotting (WB). Created in BioRender, https://BioRender.com/3udm9r4 (accessed on 20 April 2025).

A total of 100,000 cells between passage 3 and 4 were seeded per scaffold (Col I and Col I+HA), having been pre-wetted in PBS. Seeding was performed in triplicate per donor in 24-well suspension culture plates (Cellstar, Frickenhausen, Germany). Media were changed at days 3, 7 and 11. Control scaffolds without cells were treated like seeded scaffolds. On day 14, samples were collected (Figure 2). Samples were stored for different analyses in one of three ways: (1) placed in TRIzol™ reagent (Invitrogen, Waltham, MA, USA) and frozen at −80 °C; (2) placed in a cryotube and frozen at −80 °C; (3) placed in Kryofix solution (50% ethanol, 3% PEG300). The samples in Kryofix were embedded in paraffin, sectioned at 5 µm thickness and stained with hematoxylin and eosin (H&E). Images were visualized using Case Viewer 2.4 software.

Protein expression was analyzed through Western blotting using a 10% SDS-PAGE gel from all the samples. The primary antibody anti-alpha smooth muscle actin (α-SMA) was used in dilution 1:2000 (clone 1A4, A-2547, Merck) in combination with anti-GAPDH diluted 1:1000 (clone 14C10, ID218, Cell Signaling Technology, Danvers, MA, USA). As secondary antibodies, 1:10,000-diluted goat polyclonal anti-mouse IgG—IRDye 800CW-conjugated (LI-COR Biotechnology Inc., 926-32210, Lincoln, NE, USA)—and 1:15,000-diluted goat anti-rabbit polyclonal IgG antibodies—IRDye 680CW-conjugated (LI-COR Biotechnology, 926-32221)—were used. Blots were analyzed using Odyssey CLx (LI-COR).

The samples stored in TRIzol reagent were processed for RNA isolation. In short, scaffolds were crushed with a pipet tip and incubated for 20 min at room temperature, followed by brief centrifugation at 12,000× *g* at 4 °C. The supernatant was transferred to a new tube and RNA was isolated using the RNeasy mini kit (74106, QIAGEN, Hilden, Germany) and RNase-free DNase set (79254, QIAGEN). RNA yield and purity were quantified using the NanoDrop spectrophotometer (Thermos Scientific, Rockford, IL, USA). Gene expression levels were measured using reverse transcription–quantitative polymerase chain reaction (RT-qPCR), as described in Oostendorp et al. [22]. Primer sets included ACTA2, EN1, TGFB1 TGFB3, COL10A1 and COL14A1, using GAPDH and YWHAZ as reference genes (Supplementary Table S2). A normalized expression (ΔΔCq) calculation was performed according to the CFX Maestro™ software User Guide Version 1.0 (Bio-Rad), using the relative quantity of the target gene normalized to the quantities of the reference genes.

### 2.4. Statistical Analysis

Data were analyzed and visualized using GraphPad prism 10.4.0 (La Jolla, CA, USA). Regarding biochemical analyses, descriptive statistics and a one-way ANOVA with Tukey’s multiple comparison test were used. For in vitro studies, comparisons between scaffolds and cell type groups were conducted under two-way ANOVA using Tukey’s multiple comparison test, e.g., average adult vs. fetal vs. eschar in Col I and Col I+HA. For comparisons between the same donors, a paired two-tailed *t* test with a confidence level of 95% was used, e.g., Col I vs. Col I+HA with fetal cells (3 donors).

## 3. Results

### 3.1. Incorporation of HA in Collagen Scaffolds

**Figure 3:**
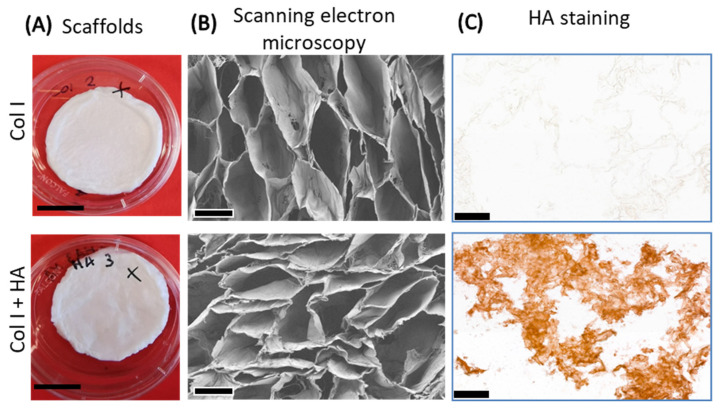
Scaffold structure and distribution of hyaluronan. Top images show Col I and bottom images show Col I+HA. (**A**) Macroscopic images of crosslinked scaffolds. (**B**) Scanning electron microscopy (SEM) shows pore morphology of collagen-only and collagen–hyaluronan scaffold cross-sections. (**C**) Immunostaining of collagen-only scaffold shows absence of hyaluronan in comparison with overall presence of hyaluronan in scaffold with HA, as stained with biotinylated HA binding protein. HA: hyaluronan. Scale bars for macroscopic images: 10 mm; SEM: 120 µm; immunostaining: 100 µm.

Porous collagen scaffolds with and without HA were prepared and characterized. Scanning electron microscopy images revealed that the covalent binding of HA slightly impacted on the morphology of the collagen scaffolds, making the pores more compact while giving them a wavy, velvety appearance (Figure 3B). Immunohistochemical staining for HA showed an even distribution in the scaffold, thereby confirming its incorporation (Figure 3C).

Table 1 gives an overview of the biochemical analysis of the scaffolds with and without HA before and after chemical crosslinking. Although the collagen scaffolds had slightly more primary amine groups per mg, the crosslinking degree was 42% for both scaffolds. Chemical crosslinking made the scaffolds more stable, seen as the denaturation temperature (Td) increased by 18 ± 7 and 17 ± 9 °C for Col I and Col I+HA, respectively.

Papain digestion was used to degrade the collagen scaffolds and quantify the amount of HA present. HA quantification showed that chemical crosslinking was necessary to capture HA, as the HA content in the crosslinked scaffolds was considerably higher than in the non-crosslinked scaffolds. As the non-crosslinked scaffolds received the same incubation steps as their crosslinked counterparts, this illustrates that the HA was partially washed out. The Col I+HA scaffolds were also evaluated in an agarose gel to estimate their molecular weight (MW, Supplementary Figure S2). The HA dissolved in water demonstrated a MW of ~800 kDa, which matches the manufacturer’s information. When HA was digested by papain, its MW decreased to ~100 kDa, similar to its size in the crosslinked Col I+HA scaffolds. It seems that the papain preparation used also affected the molecular weight of the HA, making it challenging to accurately assess HA size in the scaffolds. The non-crosslinked and untreated scaffolds with HA showed higher MWs in a range from 93 to 370 kDa. HA was not observed in the Col I scaffolds by agarose gel electrophoresis.

### 3.2. Effect of Hyaluronan-Containing Scaffolds on Macrophage Polarization

**Figure 4:**
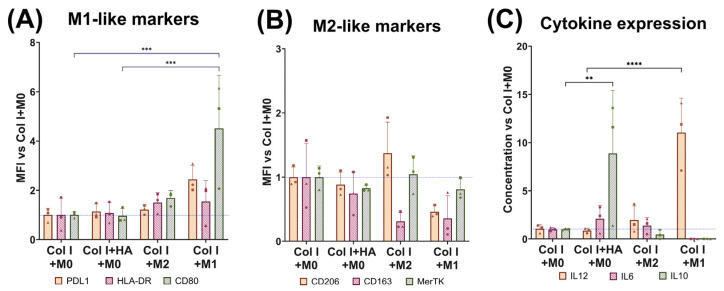
Macrophage CD markers and cytokine expression levels after 48 h of culture on collagen scaffolds. Flow cytometry for (**A**) M1-like markers for PDL1, HLA-DR and CD80 and (**B**) M2-like markers for CD206, CD163 and MerTK. (**C**) Analysis of cytokine expression of IL12, IL6 and IL10 in culture supernatant. Donors represented as follows: ● donor 1; ■ donor 2; ▲ donor 3. Comparison between groups analyzed using two-way ANOVA. ** = *p* < 0.01, *** = *p* < 0.001, **** = *p* < 0.0001.

Since the immune response has an important role in wound healing, especially in the phases of inflammation and remodeling, the effect of scaffold composition on macrophages was evaluated. Chemically crosslinked scaffolds were used for the biological analysis. M0 macrophages were seeded in Col I scaffolds ± HA, while M1 or M2 macrophages were seeded in Col I scaffolds as controls. After 2 days, the cells were analyzed by flow cytometry using the MFI of the cell surface markers (Figure 4). The expression of CD80 in the M0 macrophages was not significantly affected by the presence of HA. The control M1-like cells in the scaffolds showed significantly higher CD80 expression than the M0 macrophages in both scaffold types (Figure 4A). PDL-1 and HLA-DR were also not induced on the M0 macrophages by HA, while slightly higher, non-significant expression was seen for the M1-like phenotype. The marker profile of the M0 macrophages in scaffolds resembled the M2-like control more than the M1-like control, especially for CD206 and MerTK (Figure 4B). The expression of CD163 was low in the control with M2-like-induced macrophages, with values similar to the M1-like-induced macrophages.

Regarding cytokine expression, IL12 was statistically higher in the control with the M1 macrophages, thereby confirming their M1 subtype. IL10 levels were notably elevated in the M0 macrophages cultured on the Col I+HA scaffolds (Figure 4C). IL-6 levels were similar for the scaffolds seeded with M0 or M2 macrophages in Col I+HA and Col I, respectively.

### 3.3. Effect of Hyaluronan on Different Fibroblast Types

Fibroblasts from different sources—adult, fetal and eschar—exhibit different markers during wound healing. To investigate whether the use of HA influenced the regenerative or fibrotic responses of these cells, fibroblasts were seeded on chemically crosslinked scaffolds and characterized through histology and gene and protein expression studies.

**Figure 5:**
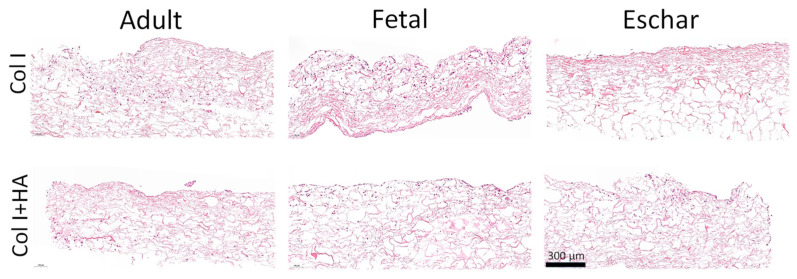
Cellular distribution in scaffolds after 14 days of culture with either adult, fetal or eschar fibroblasts using H&E staining. Collagen stained in pink; cells stained in purple. Scale bar: 300 µm.

In H&E staining, slightly more fetal fibroblasts seem to be present on the scaffolds after two weeks of culture, followed by the adult and eschar cells, which correlates with the amount of isolated RNA (Supplementary Table S3) and growth rate in 2D culture (Supplementary Figure S3). The adult and fetal cells showed slightly higher migration in both scaffold types than the eschar cells, which mostly remained on the surface (Figure 5). The fetal cells formed cellular aggregates, which may have been due to their higher proliferation rate. Cellular morphology varied somewhat between donors, regardless of the presence of HA. Overall, the fetal and adult fibroblasts had a more roundish shape, while the eschar cells were more elongated (Supplementary Figure S4). As histology only shows sections of the scaffolds, we used mRNA and protein expression to obtain more representative results from larger specimens.

**Figure 6:**
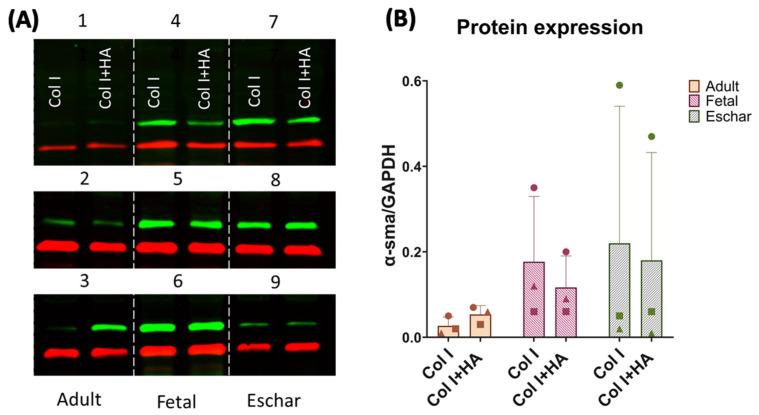
α-SMA protein expression after culturing different types of fibroblasts in scaffolds for 14 days. (**A**) Western blot results for α-SMA of human fibroblast donors 1-9. Molecular weight of α-SMA (green): 42 kDa; GAPDH (red): 36 kDa. (**B**) α-SMA/GAPDH quantification of Western blot results. Individual donors represented by different symbols as follows: ● donor 1/4/7; ■ donor 2/5/8; and ▲ donor 3/6/9.

The protein expression of α-SMA was evaluated as a marker for myofibroblast presence. α-SMA expression was not affected by the addition of HA to the Col I scaffolds (Figure 6 and Supplementary Figure S5), but it differed between fibroblast types, although not significantly. High expression of α-SMA was observed for the fetal and eschar cells, while the expression of α-SMA was very low or hardly detectable for the adult dermal cells, except for donor 3 in the HA scaffolds (Figure 6A).

**Figure 7:**
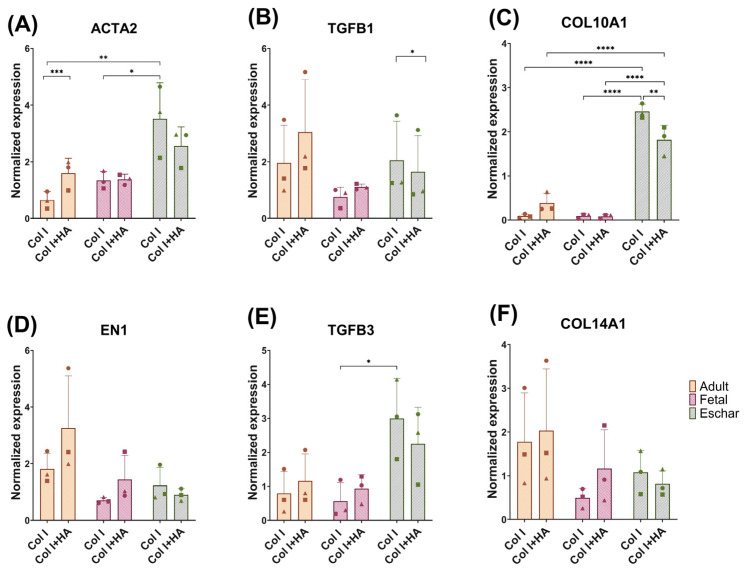
Effect of hyaluronan in collagen scaffolds on mRNA expression by adult, fetal and eschar fibroblasts after 14 days of culture. Normalized expression (ΔΔCq) of (**A**) alpha smooth muscle actin 2 (ACTA2), (**B**) transforming growth factor beta 1 (TGFB1), (**C**) type X collagen alpha 1 chain (COL10A1), (**D**) engrailed-1 (EN1), (**E**) transforming growth factor beta 3 (TGFB3) and (**F**) type XIV collagen alpha 1 chain (COL14A1). Individual donors represented by different symbols: ● donor 1/4/7; ■ donor 2/5/; ▲ donor 3/6/9. ΔΔCq calculated using relative quantity of target gene normalized to quantities of reference genes. Note: Comparison between groups analyzed using two-way ANOVA; paired *t* tests applied for comparison between same donors. * = *p* < 0.05, ** = *p* < 0.01, *** = *p* < 0.001, **** = *p* < 0.0001.

To corroborate the Western blot results at the mRNA level, RT-qPCR was performed (Figure 7). The expression of ACTA2, which encodes the protein α-SMA, was more affected by cell origin than by the presence of HA (Figure 7A), similarly to the observed protein levels in Figure 6. The expression of α-SMA in the adult cells was increased in Col I+HA, whereas its expression in the fetal and eschar cells was not significantly affected by the presence of HA. ACTA2 expression in the Col I scaffolds was increased in the eschar cells compared to the adult and fetal cells.

TGFβ1 can induce the conversion of fibroblasts into myofibroblasts, leading to fibrosis [23]. The expression of TGFB1 was slightly but significantly reduced in the eschar cells when using HA in the scaffolds, as assessed by paired testing (Figure 7B).

Another gene related to myofibroblasts is COL10A1, which is regulated by TGFβ1 [24,25]. Among all the genes analyzed in this study, this gene showed the highest expression in the eschar cells compared to the other cell types (Figure 7C). In the presence of HA, COL10A1 expression was significantly reduced in the eschar cells.

Engrailed-1 (EN1) plays an important role in the reorganization of the cytoskeleton [26,27]. Its expression is low during early pregnancy and during wound regeneration but high when scarring occurs [28]. The expression of the EN1 gene was not affected by HA being added to the scaffolds (Figure 7D).

TGFβ3 is highly expressed in fetal wounds and has been associated with skin regeneration [29]. Surprisingly, its highest expression level was observed in the eschar cells (Figure 7E), with no significant difference between the adult and fetal cells. The expression of COL14A1, associated with embryogenesis and the regulation of collagen fibrillogenesis, was similar between the adult, fetal and eschar cells in both scaffold types (Figure 7F).

## 4. Discussion

Hyaluronan (HA) is a component widely used in skin care products and in promising dermal templates to treat burn injuries, such as Hyalomatrix^®^. Many studies have focused on the effect of HA on a specific cell type, predominantly in the form of hydrogels. In this study, we created porous collagen sponges biofunctionalized with HA and showed their promising regenerative effect on macrophages and fibroblasts of different origins.

The concentration of 0.05% HA for our collagen scaffolds was selected based on the study by Uijtdewilligen et al. [3], where the highest incorporation of this glycosaminoglycan was obtained at this concentration. SEM images showed the fluffy edges of the cross-section of the scaffolds, which might be some HA coating the scaffold walls. Similar observations were made for chitosan [30] and silk [31] scaffolds supplemented with HA. Immunostaining proved that HA was distributed evenly in the scaffolds when mixed during its production. We achieved ~43 µg HA/mg scaffolds, which was twice of what has been reported [3]. Overall, the incorporation of HA into the scaffold did not modify its crosslinking degree nor its denaturation temperature, indicating a minimal indirect impact on mechanical properties [32]. The largest effect came from the chemical crosslinking, which resulted in a higher temperature being required to denature the collagen, as observed in previous studies [19].

It is well known that fetal wounds are able to regenerate free of scars and that the amount of HA in the extracellular matrix of the skin in mammals is higher during the early embryonic life stage [33,34]. Here, we mimicked this aspect of the fetal microenvironment by creating Col I+HA scaffolds and studied the subsequent response of macrophages and fibroblasts, after confirming the incorporation of HA in the collagen scaffolds.

The activation of specific macrophage phenotypes during different stages of wound healing could make the difference between proper skin remodeling and scarring [35]. We noticed that, upon seeding M0 macrophages on our scaffolds containing either Col I or Col I+HA, the macrophages started to resemble a phenotype closer to M2-like macrophages (CD80^low^, CD206^high^, CD163^high^ and MerTK^high^). This phenotype, however, differed from the control macrophages that were stimulated using IL4 and IL13 (M2-like cells) and seeded on Col I scaffolds. The M2 control was more similar to an M2a subset of macrophages (CD80^low^, CD206^high^, CD163^low^ [36]), whereas macrophages collected from the scaffolds were more similar to M2c-like macrophages (CD163^high^, CD206^high^) [37]. This was confirmed by the increased IL10 production under conditions with HA, as M2c macrophages secrete large amounts of IL10 [38]. Additionally, it is known that IL10 is essential in regenerative wound healing in fetal tissue [39] and that it can elevate HA deposition in the ECM of fetal fibroblasts [40].

Several studies have explored the effects of HA on macrophage activation, with low-MW HA promoting pro-inflammatory and angiogenic responses, while high-MW HA reduces inflammation and angiogenesis [41]. In our study, the HA within crosslinked scaffolds (~100 kDa) exhibited a reduced MW after papain digestion, but this was also seen after HA digestion alone. Interestingly, macrophage polarization in chemically crosslinked HA–collagen hydrogels is reportedly not significantly affected by the MW of the HA [42]. We observed an M2c-like macrophage profile in our Col I+HA scaffolds, and it is known that M2-like subtypes show significant CD44 upregulation, enhancing responsiveness to HA [43,44]. In this context, M2c-like macrophages contribute to wound healing by secreting IL10, which could downregulate TGFβ signaling, thereby inhibiting fibroblast-to-myofibroblast differentiation and showing promise in reducing excessive scarring [45].

Histological images showed that the number of fibroblasts in the scaffolds was somewhat higher for the fetal fibroblasts, followed by the adult and eschar cells, regardless of whether they were seeded in Col I or Col I+HA scaffolds. This may be related to a higher proliferation rate [42], as was seen during their expansion in 2D culture, and by the larger amounts of isolated RNA for fetal samples. Although small fragments of HA (6–20 monosaccharides in length) could attract fibroblasts to the wound site through CD44 signaling during the proliferation phase [46], we did not see an increased migration when using Col I+HA scaffolds, which may be due to the static culture in combination with the crosslinking of HA to collagen, which could restrict the available binding sites.

Scarring is caused by a fibrotic response during wound healing and is characterized by high α-SMA expression in fibroblasts [47]. Our findings revealed that fetal fibroblasts produced more α-SMA than adult fibroblasts at the protein level when cultured in collagen scaffolds, consistent with studies using these two cell types on dermal templates (Novomaix) [48]. However, α-SMA expression was not affected by the presence of HA. The eschar fibroblasts, which exhibited a myofibroblast-like profile [13], showed an approximately two-fold increase in COL10A1 mRNA levels compared to the adult and fetal fibroblasts. This response was decreased in the presence of HA. While ACTA2, EN1 and TGFB1 are regarded as fibrotic genes [26], they play crucial roles in embryogenesis and skin wound healing [49,50,51]. COL10A1 may serve as a potential marker to identify scar-associated cells like eschar fibroblasts [52,53], as its expression was notably higher in these cells. This could aid in distinguishing fibroblasts involved in excessive scarring in order to develop new strategies to reduce these cells, ultimately contributing to improved wound healing and reduced scarring in patients.

Skin regeneration has been observed during fetal wound healing [54]. Therefore, we investigated COL14A1 and TGFB3, as these genes have been related to regeneration and embryogenesis [55]. However, we did not observe a high expression of these genes in fetal cells, irrespective of the use of HA in scaffolds. Fibroblasts are mechanosensitive, which was shown by the reduced α-SMA expression when the cells were exposed to a softer 3D environment compared to a 2D one in plastic [56]. It is possible that our fetal fibroblasts did not upregulate TGFB3 or COL14A1 expression because they were present in a 3D scaffold and not surrounded by a fibrotic environment [57], which would otherwise activate fibrillogenesis [55]. Overall, the largest differences were related to fibroblast origin rather than the incorporation of HA.

While our study provides valuable insights about the potential of HA to improve wound healing, certain considerations might enhance the applicability of these findings. We observed some variability in expression levels across cell donors in our in vitro studies, although a consistent trend was noted in their expression profiles. To address this, we recommend increasing the cell density to 200,000/cm² for fibroblasts and macrophages, and expanding the number of donors to increase the heterogeneity [58]. Since normal adult fibroblasts may not accurately reflect the responses observed in a fibrotic environment, fibrotic cells like eschar fibroblasts or wound models are better suited for such studies. For future experiments, it would be interesting to seed M2-like and M1-like stimulated macrophages on Col I+HA to observe if their phenotypes maintain or switch.

## 5. Conclusions

In conclusion, by chemical crosslinking we successfully incorporated HA into collagen scaffolds, which may be used as a prototype for dermal substitutes in the future. Our results demonstrated that Col I+HA scaffolds increased IL10 cytokine expression, a marker of M2c-like macrophages. Additionally, the scaffolds containing HA reduced the expression of scarring-associated genes (TGFB1, COL10A1) in primary eschar fibroblasts, further supporting their potential to promote a reparative rather than fibrotic response.

## Figures and Tables

**Table 1:** Characteristics of type I collagen scaffolds with and without hyaluronan. N = 3 ± SD.

Scaffold Type	Amine Group Content (nmol/mg Scaffold)		Denaturation Temperature Td (°C)		Hyaluronan Content (µg/mg Scaffold)	
Crosslinked	No	Yes	No	Yes	No	Yes
Col I	361 ± 49	206 ± 19	59 ± 1	78 ± 0	1 ± 1	1 ± 1
Col I+HA	341 ± 36	197 ± 23	58 ± 1	76 ± 0	19 ± 4	43 ± 2

## Data Availability

The original contributions presented in this study are included in the article/Supplementary Materials. Further inquiries can be directed to the corresponding author.

## Supplementary Materials

The following supporting information can be downloaded at: https://www.mdpi.com/article/10.3390/jfb16050167/s1, Figure S1: Representative gating strategy to select CD45+ macrophages; Figure S2: Molecular weight of hyaluronan decreased after digestion with papain and crosslinking with collagen scaffolds using an agarose gel stained with Stains-All; Figure S3: Microscopic images displaying higher proliferation of fetal fibroblasts in a tissue culture flask, prior to seeding on the scaffolds; Figure S4: H&E staining of scaffolds after 14 days of culture with fibroblasts of different origins; Figure S5: α-SMA protein expression after culturing different types of fibroblasts in scaffolds for 14 days; Table S1: Antibody panels used to characterize macrophages by flow cytometry; Table S2: Primer sequences for quantitative polymerase chain reaction; Table S3: RNA quantification in fibroblasts seeded on scaffolds after 14 days of culturing.
