# Supplementary material for: Effect of Hyaluronan in Collagen Biomaterials on Human Macrophages and Fibroblasts In Vitro
Source: J Funct Biomater. 2025 May 8;16(5):167. doi: 10.3390/jfb16050167 (PMC12112233; doi:10.3390/jfb16050167)
Supplement: Supplementary file 1 [file jfb-16-00167-s001.zip › jfb-3576587-supplementary.pdf]

Supplementary material

Effect of Hyaluronan in Collagen Biomaterials on Human Macrophages and Fibroblasts In Vitro

Nancy Avila-Martinez [1], Maren Pfirrmann [1], Madalena L. N. P. Gomes [2,3,4,5], Roman Krymchenko [1], Elly M. M. Versteeg [1], Marcel Vlig [5], Martijn Verdoes [1,6], Toin H. van Kuppevelt [1], Bouke K. H. L. Boekema [3,4,5] and Willeke F. Daamen [1,*]

[1] Department of Medical BioSciences, Research Institute for Medical Innovation, Radboud university medical center, 6525 GA, Nijmegen, The Netherlands
[2] Department of Pathology, Amsterdam University Medical Center (AUMC), location AMC, 1081 HV, Amsterdam, The Netherlands
[3] Tissue Function and Regeneration, Amsterdam Movement Sciences Research Institute, 1081 HV, Amsterdam, The Netherlands
[4] Department of Plastic, Reconstructive and Hand Surgery, Amsterdam University Medical Center (AUMC),, location AMC, 1081 HV, Amsterdam, The Netherlands
[5] Alliance of Dutch Burn Care, Burn Research Lab, 1941 AJ, Beverwijk, The Netherlands
[6] Department of Immunology, Leiden University Medical Center (LUMC), 2333 ZA, Leiden, The Netherlands
* Correspondence: willeke.daamen@radboudumc.nl

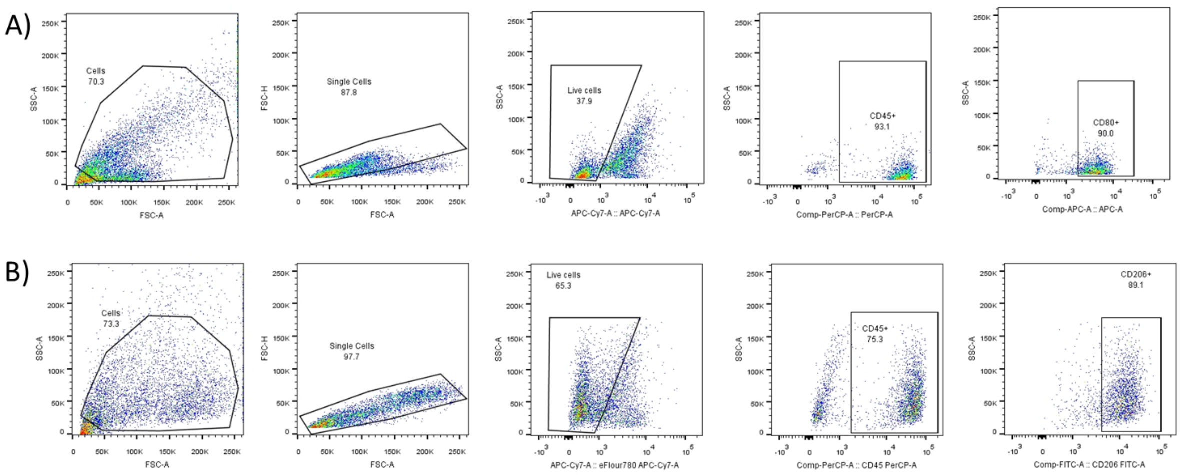

Figure S1. Representative gating strategy to select CD45⁺ macrophages. A) CD80⁺ in sample Col I+M1-like macrophages and B) CD206⁺ in sample Col I+M2-like macrophages.

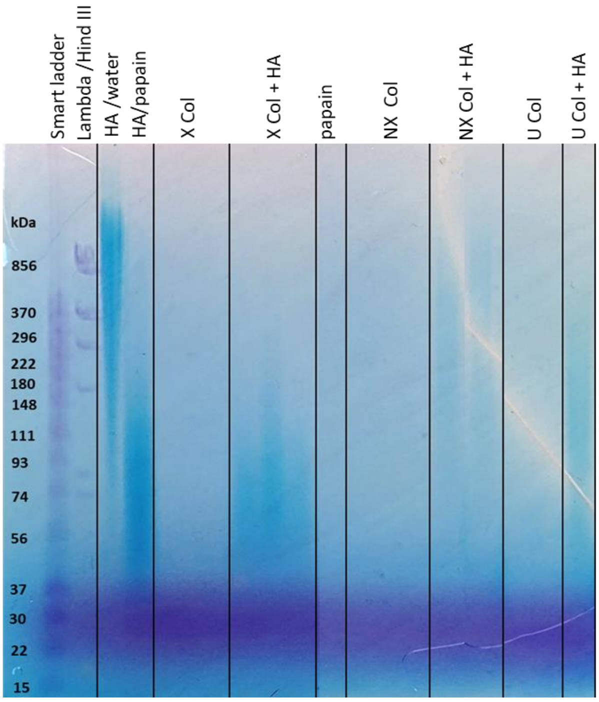

Figure S2. Molecular weight of hyaluronan decreased after digestion with papain and crosslinking with collagen scaffolds using an agarose gel stained with Stains-All. Papain-digested scaffolds from different batches were applied to the gel for the following conditions: crosslinked (X), non-crosslinked (NX) and untreated (U) made by type I collagen (Col I) or type I collagen + hyaluronan (Col I +HA). HA dissolved in demineralized water (HA/water) and digested in papain (HA/papain) were included as controls. DNA ladders were used for the estimation of the molecular weight and converted from pair base to polysaccharide kDa.

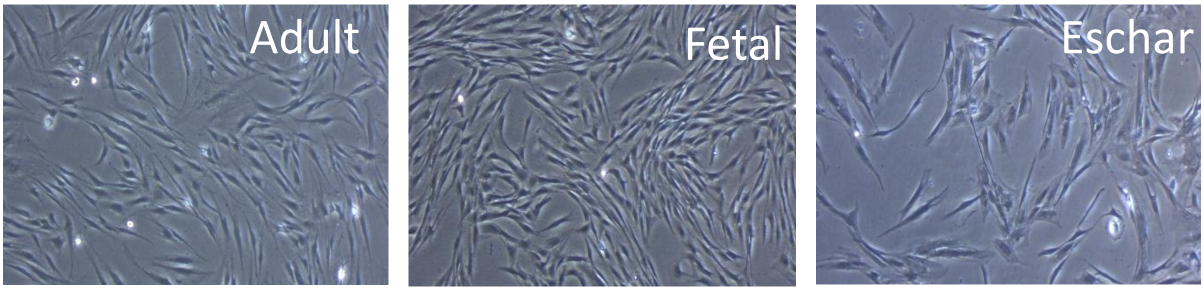

Figure S3. Microscopic images displaying higher proliferation of fetal fibroblasts in a tissue culture flask, prior to seeding on the scaffolds. 0.9 x 10^6 cells/T175 flask were seeded in each condition and photos were captured after 7 days of culture. Original magnification 10x.

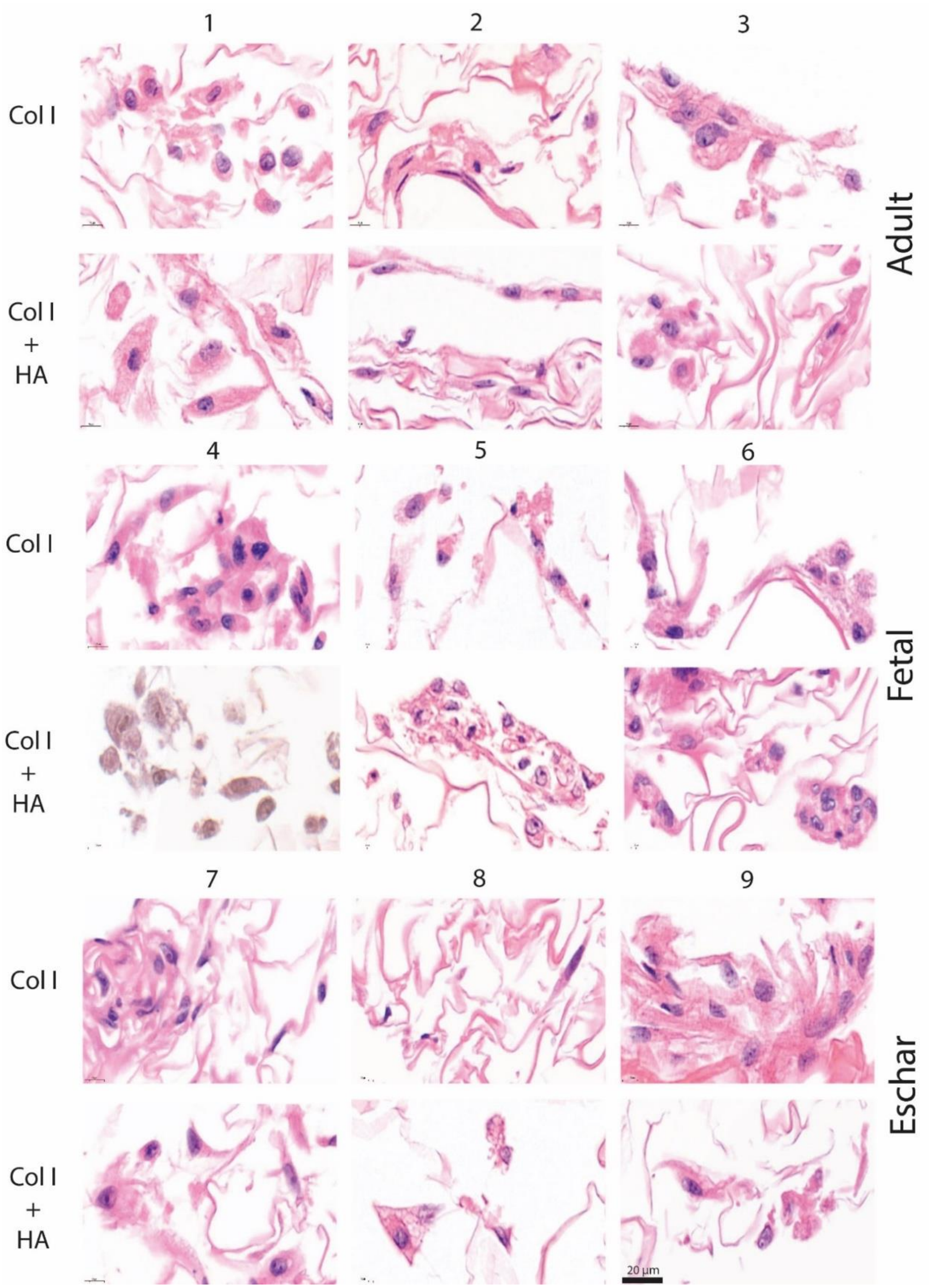

Figure S4. H&E staining of scaffolds after 14 days of culture with fibroblasts of different origins. Adult (donor 1, 2, 3), fetal (donor 4, 5, 6) and eschar (donor 7, 8, 9) fibroblasts were used. Scale bar is 20 µm.

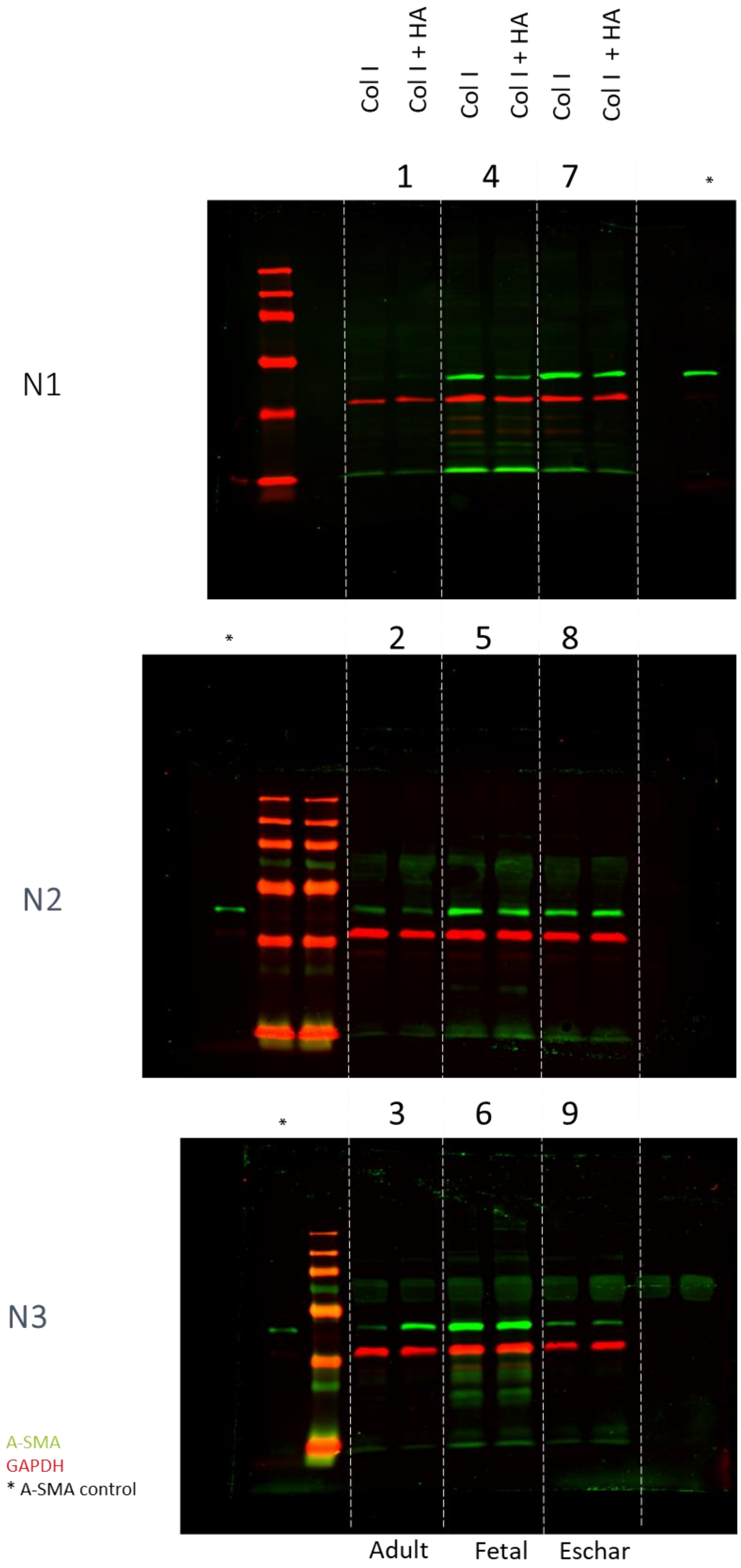

Figure S5. α-SMA protein expression after culturing different types of fibroblasts in scaffolds for 14 days. A) Western blot results for α-SMA of human fibroblast donors 1-9. Molecular weight of α- SMA (in green) is 42 kDa and GAPDH (in red) is 36 kDa.

Table S1. Antibody panels used to characterize macrophages by flow cytometry.

Panel 1			
**Label/fluorophore**	**Surface marker**	**Dilution**	**Cat. No., Company**
BV421	CD163	1:40	562643, BD Biosciences
FITC	CD206	1:40	551135, BD Biosciences
APC	CD80	1:20	305220, BioLegend
PeCy7	MerTK	1:50	367610, BioLegend
PerCP	CD45	1:20	304026, BioLegend
**Panel 2**			
**Antibody**	**Surface marker**	**Dilution**	**Description**
PE	PDL1	1:30	557924, BD Biosciences
BV510	HLA-DR	1:20	307646, BioLegend
PerCP	CD45	1:20	304026, BioLegend

Note: Companies location BD Biosciences, Franklin Lakes, NJ, USA and BioLegend, San Diego, CA, USA.

Table S2. Primer sequences for quantitative polymerase chain reaction.

**Gene**	**Name**	**Sequence**
GAPDH	Glyceraldehyde-3-phosphate dehydrogenase	5′ TGG GTG TGA ACC ATG AGA AG 3′<br>3′ AGT TGT CAT GGA TGA ACC TTG G 5′
YWHAZ	Tyrosine 3-monooxygenase / Tryptophan 5-monooxygenase activation protein zeta	5′ CAT CTT GGA GGG TCG TCT CA 3′<br>3′ ACT TTG CTC TCT GCT TGT GAA 5′
ACTA2	Alpha smooth muscle actin 2	5′ CCG ACC GAA TGC AGA AGG A 3′<br>3′ ACA GAG TAT TTG CGC TCC GAA 5′
EN1	Engrailed-1	5′ TGA CTC GCA GCA GCC TCT CGT 3′<br>3′ AAC GTG TGC AGT ACA CCC AGG C 5′
TGFB1	Transforming growth factor beta 1	5′ CGC GTG CTA ATG GTG GAA A 3′<br>3′ TGT GTG TAC TCT GCT TGA ACT TGT CA 5′
TGFB3	Transforming growth factor beta 3	5′ CAA TTA CTG CTT CCG CAA CTT G 3′<br>3′ GAT CCT GTC GGA AGT CAA TGT AGA 5′
COL10A1	Type X collagen alpha 1 chain	5′ GGG AGT GCC ATC ATC GAT CT 3′<br>3′ CCA TTT GAC TCG GCA TTG GG 5′
COL14A1	Type XIV collagen alpha 1 chain	5′ TGT GGA TGA CTT TGA CGC CT 3′<br>3′ GTT GCT GAT GCT GTT TCG CA 5′

Table S3. RNA quantification in fibroblasts seeded on scaffolds after 14 days of culturing.

Nucleic acid concentration (ng/µl)	Adult	Fetal	Eschar
Col I	121 ± 18	223 ± 64	100 ± 37
Col I + HA	125 ± 53	206 ± 40	126 ± 70
